# Genome-wide mapping of stress-responsive lncRNA, uc.104, reveals the chromatin-mediated regulation of stress and plasticity-related genes in the hippocampus of chronic restraint rats

**DOI:** 10.1186/s13041-026-01304-3

**Published:** 2026-04-19

**Authors:** Anuj K. Verma, Bhaskar Roy, Kevin Prall, Ellie Hulwi, Yogesh Dwivedi

**Affiliations:** Department of Psychiatry and Behavioral Neurobiology, Heersink School of Medicine, University of Alabama at Birmingham, SC711 Sparks Center 1720 7th Avenue South, Birmingham, AL 35294 USA

**Keywords:** lncRNA, Hippocampus, Depression, Chromatin, Gene regulation

## Abstract

Chronic stress significantly impacts hippocampal function through transcriptional and epigenetic mechanisms. While the roles of lncRNAs in stress-related transcriptional and epigenetic regulation have recently been recognized, their genome-wide functions controlling the transcriptional network remain largely unclear. Evidence indicates that the lncRNA uc.104 is involved in stress responses; however, its genome-wide chromatin interactions and gene regulatory effects are yet to be explored. To examine this, we combined chromatin isolation by RNA purification sequencing (ChIRP-seq) and RNA sequencing (RNA-seq) in the hippocampus from handled control and chronic restraint stress (CRS) rats. ChIRP-seq identified 6,664 uc.104 binding peaks under CRS, including 6,517 enriched and 149 reduced. Many peaks were mapped to intronic and promoter-proximal regions of protein-coding genes. Integration of ChIRP-seq with RNA-seq data revealed 1,839 differentially expressed genes associated with uc.104 binding sites, with 106 high-confidence overlaps. Several genes (Gabra3, Htr7, Irs1, Gpr37, Clu, Hspa1b, Ppp3r2, Nfasc, Pcdhac2, and Cysltr2) identified as regulatory targets of uc.104, have been directly implicated in stress responses, synaptic plasticity, and neuroinflammation. Gene ontology and Synapse GO (SynGO) analyses revealed significant enrichment for processes involving dendritic spine formation, synapse organization, and pre- and postsynaptic signaling. Protein–protein interaction analysis identified hub genes, including EGFR, CDC42, IGF1R, CTNNB1, CALM1, CALM3, POLR2A, MDM2, TBP, and CSNK1E, several of which have been linked to stress-responsive pathways. Together, our findings reveal that uc.104 binding to chromatin near stress- and synapse-related genes may act as a regulator of stress-responsive transcriptional networks in the hippocampus. By linking uc.104 occupancy to stress and synaptic responsive genes, this study highlights uc.104 as a potential mediator of stress-induced hippocampal malfunctions.

**Supplementary Information:**

The online version contains supplementary material available at 10.1186/s13041-026-01304-3.

## Introduction

Long noncoding RNAs (lncRNAs) play key roles in various cellular activities by regulating gene expression output [1, 2]. Increasing insights into their diverse mechanisms of action have firmly established them as the central regulatory hubs of gene expression, and their dysregulation can critically contribute to cellular and molecular pathologies [3]. Recently, they have gained significant recognition for their role in regulating brain function and exhibiting substantial changes in many neuropsychiatric conditions [4, 5]. Stress is a powerful environmental factor that can induce widespread changes in brain structure and function [6]. Moreover, prolonged exposure of the brain to chronic stress is associated with changes in gene expression [7]. As a result, many critical neurobiological functions, including neurogenesis and plasticity, are largely affected by these changes [8]. The hippocampus, a vital brain region involved in learning, memory, and emotional regulation, has been shown to be particularly vulnerable to stress-induced changes at the cellular plasticity level [9]. Data from preclinical and postmortem studies indicate that chronic stress leads to dendritic retraction, synaptic loss, and altered neurogenesis in the hippocampus [10]. Interestingly, all these changes are accompanied by extensive transcriptional and epigenetic remodeling of gene function, making the hippocampus one of the brain regions significantly impacted by the alterations highlighted above [7, 11].

While the transcriptional changes of protein-coding genes in response to chronic stress have been extensively characterized, the role of noncoding RNAs in mediating these changes remains less well understood [12]. LncRNAs, defined as transcripts longer than 200 nucleotides with limited protein-coding potential, have emerged as key regulators of gene expression via chromatin dynamics [13]. LncRNAs can function in cis or trans to regulate gene transcription through diverse mechanisms, including recruitment of chromatin-modifying complexes, modulation of chromatin looping, and interactions with transcription factors [2]. Recently, a growing body of evidence from our and other labs suggests that lncRNAs are dynamically regulated in the brain in response to stress and contribute to stress-induced alterations in neuroplasticity [14–17]. Transcriptomic studies have also identified lncRNA that that are differentially expressed in brain regions such as the prefrontal cortex and hippocampus following chronic stress paradigms [16, 17]. These lncRNAs have been implicated in processes such as synaptic plasticity, neuroinflammation, and glucocorticoid receptor signaling, all of which are critical for the brain’s maladaptation to stress [18, 19].

Chromatin Isolation by RNA Purification (ChIRP) coupled with next-generation sequencing (ChIRP-seq) has recently enabled comprehensive genome-wide mapping of lncRNA occupancy on chromatin [20]. This is a state-of-the-art method for analyzing the ability of lncRNAs to act as scaffolds for chromatin regulators, including the Polycomb repressive complex 2 (PRC2), SWI/SNF complexes, and histone acetyltransferases [21]. By tethering these complexes to specific genomic loci, lncRNAs can influence histone modifications and DNA accessibility, thereby regulating transcriptional outputs [22, 23]. To investigate the functional role of lncRNA in chronic stress, in this study, we focused on assessing the potential of uc.104, a key lncRNA involved in chromatin modulation that we previously found to be linked to stress response regulation [14]. In this study, we demonstrated the role of uc.104 in stress susceptibility using the learned helplessness paradigm. Differential expression analysis following transcriptome-wide expression revealed significant changes in uc.104 in the hippocampus of LH rats. This was one of the most highly expressed lncRNAs in the LH rat hippocampus. Given the remarkable response to chronic stress, uc.104 is a suitable candidate for further investigation of its regulatory landscape in stress-related pathology. In our current study, we examined the corresponding mRNA expression profile to understand the influence of uc.104-mediated chromatin changes on gene expression under chronic stress. Utilizing a rat model of chronic stress, we investigated lncRNA probe-based immunoprecipitation of chromatin fragments (ChIRP-seq) followed by next-generation sequencing in rat hippocampal tissue. The outcome of ChIRP-seq experiments delineates the potential interaction of stress-responsive lncR-uc.104 with target gene promoters, thereby modulating their expression landscape in a stress-induced, tissue-specific environment. By integrating ChIRP-seq and RNA-seq data, we aim to elucidate how stress-associated lncR-uc.104 modulate chromatin occupancy and regulate hippocampal gene networks involved in stress adaptation and pathology. This also provides novel insight into the role of this lncRNA in stress-induced molecular pathogenesis such as major depression.

## Materials and methods

All animal experiments adhered to the National Institutes of Health (NIH) guidelines for the care and use of laboratory animals and received approval from the University of Alabama at Birmingham’s Institutional Animal Care and Use Committee (IACUC).

### Animals and chronic restraint stress paradigm

Adult male Sprague-Dawley rats (8–10 weeks old, weighing 250–300 g) were obtained from Envigo (formerly Harlan Sprague-Dawley Laboratories, Indianapolis, IN, USA) and housed in standard laboratory conditions (12-hour light/dark cycle, 22 ± 2 °C, food and water ad libitum). Animals were randomly assigned to a handled control (*n* = 6) or chronic restraint stress (CRS) group (*n* = 6).

Chronic Restraint Stress (CRS) was induced by placing rats individually in clear acrylic tubes (20 cm long, 6.35 cm internal diameter, air vents in the cap and along the tube) with the tail extending from the rear of the tube. The cap was placed inward enough to prevent the rat from moving forward or backward inside the tube. Rats were restrained for 2 h/day for 14 consecutive days [24]. Control animals were handled similarly but were not subjected to restraint. Restraint protocol was followed during the light cycle (08:00 to 12:00). Our sample size justification is supported by prior work [25], which identified 57 differentially expressed ncRNAs in the hippocampus of stressed rats, with several showing highly significant differences (*p* < 0.0001) using only 7–8 animals per group. Such findings indicate large effect sizes. Consistent with this, our power analysis (α = 0.05, 80% power) showed that 6 animals per group were sufficient to detect similarly large effects (Cohen’s d ≈ 1.6–2.0).

### Hippocampal tissue collection and RNA extraction

Twenty-four hours after the final restraint session, rats were anesthetized using an isoflurane vaporizer; isoflurane was delivered through inhalation at 5% mixed with oxygen until the animal was fully unconscious. Anesthesia was maintained until euthanasia via bilateral thoracotomy. Blood was drawn via thoracotomy and cardiac puncture into EDTA tubes. The whole blood was centrifuged at 1400 rpm for 15 min at 4°C to separate the plasma as shown previously [26]. Rats were decapitated; the brains were rapidly removed; bilateral hippocampi were dissected on ice, snap-frozen in liquid nitrogen, and stored at − 80°C until use. Total RNA was extracted using TRIzol reagent (Invitrogen, USA) following the manufacturer’s protocol, followed by DNase I treatment (Qiagen RNeasy Kit) to eliminate genomic DNA contamination [27]. RNA concentration and purity were measured using a Nanodrop spectrophotometer (A260/A280 ratio), and integrity was assessed using denaturing agarose gel electrophoresis by evaluating 28 S and 18 S rRNA bands. Additionally, the Agilent 2100 Bioanalyzer was used to determine the RNA integrity number, and only samples with RIN ≥ 7.5 were used for subsequent experiments [28]. CORT levels in plasma, retrieved at the time of sacrifice, were quantified using an enzyme-linked immunosorbent assay following the manufacturer’s instructions (Enzo Life Sciences, ADI-901-097).

### Expression validation of stress-induced select lncRNAs in the hippocampus of CRS rats

Relative quantification of select lncRNA transcripts was determined following the ∆∆Ct method using the first-strand cDNA synthesized from total RNA. For 1st strand cDNA synthesis, the random hexamer-based priming method was followed. Later, the synthesized cDNA was used for qPCR quantification with target-specific primer sequences as provided in Table S1. Fold change analysis was performed using U6 as a normalizer, and results were presented as ± SEM. Statistical significance was determined by an independent-sample t-test.

### Chromatin isolation by RNA purification sequencing (ChIRP-seq)

To identify genome-wide chromatin-binding sites of selected lncRNAs in the hippocampus, ChIRP-seq was performed as described earlier with some modifications [29].

**Probe design and validation**: Biotinylated antisense DNA oligonucleotide probes (20-mer, tiling across the full length of each candidate lncRNA) were designed using Stellaris Probe Designer and synthesized (Integrated DNA Technologies, USA). The specificity of the probes was confirmed in silico to minimize off-target binding.

**Chromatin preparation**: Hippocampal tissue was crosslinked with 1% formaldehyde for 10 min at room temperature, then quenched with 0.125 M glycine. Crosslinked tissue was homogenized and sonicated to obtain chromatin fragments of 200–500 bp (confirmed on agarose gel). Chromatin lysates were hybridized overnight at 37 °C with probe sets targeting specific lncRNAs. Streptavidin-coated magnetic beads were used to capture RNA-associated chromatin complexes, which were then aliquoted into two volumes for downstream assays.

**ChIRP DNA preparation**: After stringent washing of beads: biotin-probes: RNA: chromatin adducts, DNA was eluted, reverse crosslinked, and purified using a phenol-chloroform extraction. Sequencing libraries were prepared with the NEBNext Ultra II DNA Library Prep Kit (New England Biolabs, USA) and sequenced on an Illumina NovaSeq 6000 platform (~ 30 million 150 bp paired-end reads per sample).

**Data processing**: Raw reads were quality-checked using FastQC and trimmed with Trimmomatic. Reads were aligned to the rat reference genome (Rnor_6.0) using Bowtie2. Peak calling was performed with MACS2 (*p* < 0.05), and peaks were annotated using HOMER and ChIPseeker to identify genomic features (promoters, enhancers, gene bodies) associated with lncRNA binding [30, 31]. Differential binding of uc.104 across the genome between control and CRS rats was processed in the R environment using a custom script and expressed as peak enrichment or depletion. In our analysis, raw reads were quality-checked using FastQC and trimmed with Trimmomatic. Reads were aligned to the rat reference genome (Rnor_6.0) using Bowtie2. Peak calling was performed with MACS2 (*p* < 0.05) software, and peaks were annotated using HOMER and ChIPseeker to identify genomic features (promoters, enhancers, gene bodies) associated with lncRNA binding to the promoter elements. Differential binding analysis of uc.104 across the genome between control and CRS rats was performed using the getDiffExpression.pl script from HOMER, and the output was further processed in R using a custom script for downstream analysis and summarization of peak enrichment or depletion. Duplicate rows with identical values across the data points were removed. The peaks were classified based on log2FC values, where log2FC ≥ 0 indicates higher peak enrichment in CRS rats compared to control, and log2FC ≤ 0 indicates less peak enrichment in CRS rats compared to control. Significantly enriched peaks were defined as those showing log2FC ≥ 1 and *p* ≤ 0.05 in the CRS group of rats. On the other hand, significantly less enriched peaks were defined with log2FC ≤ -1. With that sorting strategy, differentially enriched peaks were separated into up-regulated and down-regulated peaks in CRS rats.

**Replicate concordance analysis**: To assess reproducibility between ChIRP-seq samples, peak-binding matrices across replicates were converted to binary peak presence or absence based on normalized binding values. Pairwise peak overlap between samples was then quantified using the Jaccard similarity index. Hierarchical clustering based on the resulting similarity matrix was visualized as a heatmap to evaluate concordance among replicates.

### ChIRP-RNA sequencing

**ChIRP-RNA preparation**: A fraction of the adduct prepared in the above-described process was resuspended in 10× the original volume of RNA elution buffer (Tris 7.0, 1% SDS) and boiled for 15 min for reverse crosslinking, followed by TRIzol extraction. Eluted RNA was subject to library preparation for mRNA sequencing.

**RNA sequencing (RNA-seq) and analysis**: Parallel transcriptomic profiling of ChIRP-RNA was performed to identify stress-induced changes in gene expression. Ribosomal RNA was depleted using the Ribo-Zero Gold Kit (Illumina, USA), and strand-specific RNA libraries were constructed using the NEBNext Ultra II Directional RNA Library Prep Kit (New England Biolabs, USA). Sequencing was performed on an Illumina NovaSeq 6000 platform to a depth of ~ 50 million 150 bp paired-end reads per sample. Quality control and trimming were conducted using FastQC and Trimmomatic. Cleaned reads were aligned to the rat genome (Rnor_6.0) with HISAT2. Transcript assembly and quantification were performed using StringTie. Differential expression analysis was conducted using DESeq2, with significance defined as p-value ≤ 0.05 and |log2 fold change| ≥ ±1.

### In-silico gene proximity analysis and integration of ChIRP-seq and RNA-seq data

To investigate the potential regulatory relationship between uc.104 binding sites and target mRNA gene promoters, a proximity analysis was performed using genomic coordinates derived from ChIRP-seq (uc.104 occupancy) and RNA-seq (differentially expressed genes) datasets generated in this study. Binding peaks for uc.104 were annotated relative to protein-coding genes, with a particular focus on promoter-proximal regions (± 2 kb from transcription start site, TSS) and extended gene-associated regions (± 25 kb).

Spatial relationships between uc.104 peaks and gene loci were assessed using Bedtools, with the bedtools closest command applied to identify the nearest protein-coding gene for each binding peak. Additional analyses were performed in R using the GenomicRanges package to detect overlaps between uc.104 binding sites and annotated gene features, including promoters, introns, and intergenic regions, and to determine the nearest-neighbor distances [32]. To strengthen the interpretation, proximity results were integrated with mRNA expression data from RNA-seq. Genes were classified according to both their spatial association with uc 0.104 binding sites and their transcriptional regulation under CRS conditions.

### Functional prediction of target genes intersected by uc.104 binding peaks

Genes associated with lncRNA ChIRP-seq peaks were intersected with differentially expressed genes (DEGs) from RNA-seq to identify putative direct targets of stress-associated lncRNAs. Functional enrichment analyses (Gene Ontology and KEGG pathways) were performed using DAVID and GSEA to identify biological processes and pathways enriched among lncRNA target genes. IncRNA–gene regulatory networks were constructed using a custom R script and visualized with the Cytoscape plugin to identify potential regulatory relationships in the hippocampus under CRS.

## Results

### Validity of the CRS rat model

The validity of the chronic restraint stress (CRS) paradigm employed in this study is supported by previously published data from our laboratory [33]. We demonstrated that CRS produces a robust physiological stress response, as evidenced by significantly elevated plasma corticosterone concentrations (*p* = 0.022, F = 8.799, t=-2.315, df = 10) in stressed rats (15,172.997 pg/mL) compared with controls (5,540.943 pg/mL). This elevated plasma corticosterone level confirms activation of the hypothalamic–pituitary–adrenal axis.

### Expression analysis of select lncRNAs in hippocampus of CRS rats

**Fig. 1:**
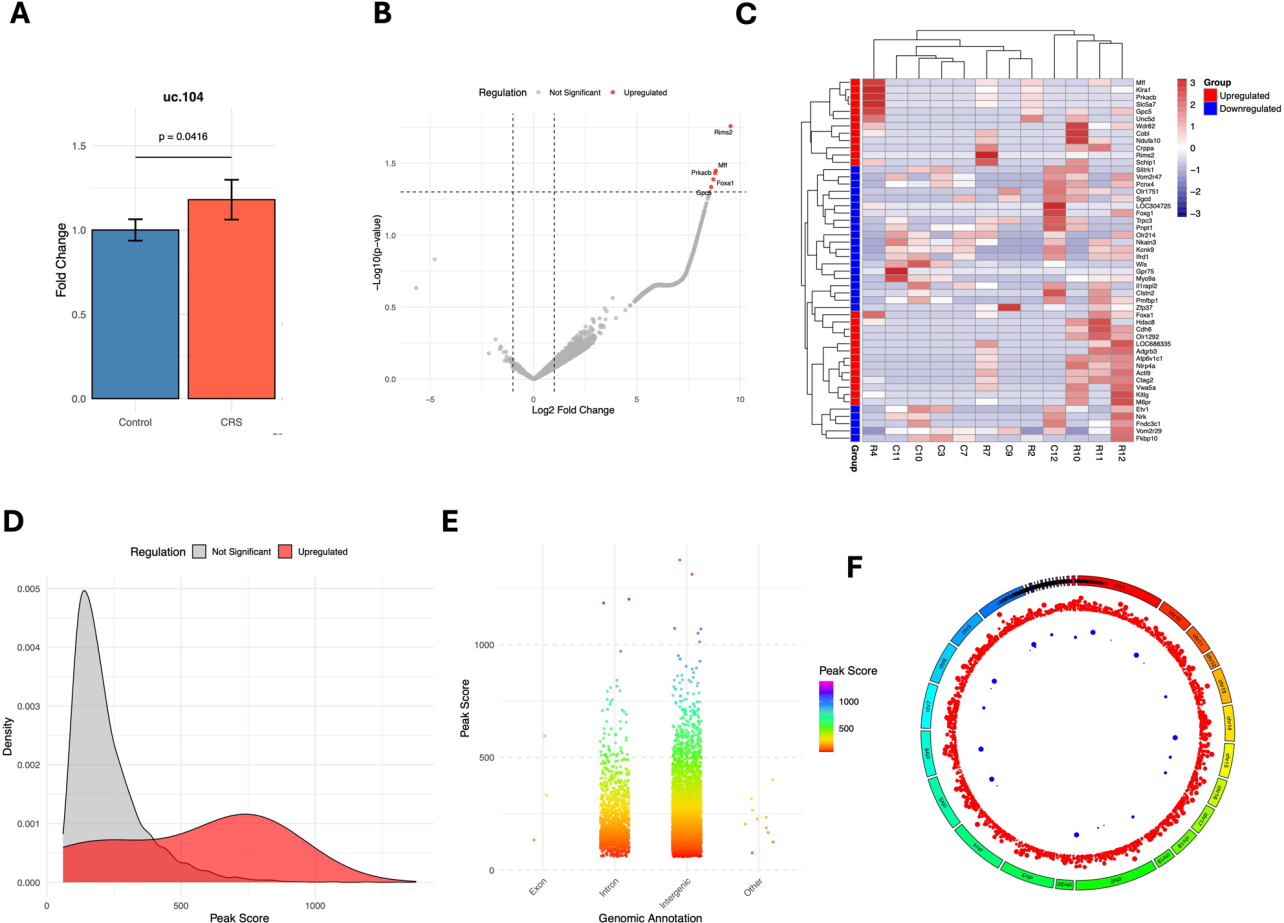
Differential regulation of select lncRNAs and genome-wide analysis of uc.104 chromatin binding in the rat hippocampus under chronic restraint stress (CRS). (**A**) In vivo differential expression profile of lncRNAs uc.104 in the hippocampus of CRS rats relative to control group. Relative transcript abundance of uc.104 was normalized to U6 and presented as expression fold change by comparing CRS with control rats. Values are presented as ± SEM and statistical differences determined by independent sample ‘t’ test (*n* = 10 in control and 9 in CRS). (**B**) Volcano plot of uc.104 binding peaks between CRS and control groups. Each dot represents a peak, with enriched peaks shown on the right and reduced peaks on the left. (**C**) Heatmap of the top 50 differentially bound genomic regions. Rows represent loci and columns represent samples, with higher occupancy shown in red and reduced occupancy in blue. (**D**) Density plot of peak score distributions across categories. Curves are colored according to binding status, with enriched peaks (red), depleted peaks (blue), and non-significant peaks (grey). (**E**) Bar plot showing genomic distribution of uc.104 peaks across promoter-proximal (± 2 kb of TSS), intronic, and intergenic regions. (**F**) Circos plot depicting chromosomal localization of uc.104 peaks. Chromosomes are represented as rainbow-colored ideograms, with enriched peaks indicated by red scatter points and depleted peaks by blue scatter points; larger points correspond to higher peak scores

We selected uc.104 as one of the top-most lncRNAs responsive to stress-associated changes in the hippocampus of the LH rat model based on our previous report.^14^ We confirmed the expression of uc.104 as the stress-responsive lncRNA in our CRS rat model. Using qPCR analysis, we found a significant increase (*p* = 0.041) in the expression of uc.104 in CRS rats relative to the control group. The qPCR expression data are presented with barplot in Fig. 1A.

### Genome-wide mapping of lncRNA-uc.104 vs. chromatin interactions by ChIRP-seq

To investigate the genome-wide chromatin occupancy of stress-responsive lncRNA uc.104 in the hippocampus, ChIRP-seq was performed using biotinylated probes targeting uc.104 in hippocampal tissue from control and CRS rats. The sequencing yielded a high-quality dataset, with each ChIRP-seq library providing confident mapping to the rat reference genome (Rnor_6.0). This underscores the specificity of the probes and validates the robustness of our ChIRP method for probing lncRNA binding to its respective chromatin regions.

To assess stress-induced changes in lncRNA-chromatin interactions, differential binding analysis was performed between control and CRS groups. For uc.104, CRS was associated with 6517 stress-enriched peaks (log2FC ≥ 0) and 149 stress-reduced peaks (log2FC ≤ 0), suggesting a highly dynamic genomic occupancy landscape in response to chronic stress. Interestingly, after applying statistical significance (*p* ≤ 0.05), we found five highly stress-enriched peaks near the sites of protein-coding genes (Gpc5, Rims2, Prkacb, Foxa1 and Mff) with log2FC > 8.5. To visualize the differential distribution pattern of enriched and depleted peaks between CRS and control rats, a volcano plot was generated, and the results are depicted in Fig. 1B. Additionally, a heatmap was prepared for the top 50 differentially bound genomic regions (top 25 up- and 25 downregulated peak regions, Fig. 1C). In this heatmap, each row corresponds to a genomic locus, and each column represents a sample from either the control or CRS group. Higher occupancy is depicted in red, while reduced occupancy is shown in blue. The heatmap highlights distinct clusters of genomic regions with stress-dependent gain or loss of lncRNA binding. The chromosomal annotation, along with its genomic coordinates, is provided in Table 1.

**Table 1:** Top 50 differential peaks bound with uc.104 lncRNA in CRS rat hippocampus

Chr	Start	End	Strand	Peak Score	Annotation	Gene Name	Log2 FC	*p*-value
chr7	78387217	78387367	+	87.6	Intron (NM_053945, intron 14 of 28)	Rims2	9.532403311	0.017
chr9	90,512,085	90512235	+	286	Intergenic	Mff	8.816752844	0.035
chr2	252665484	252665634	+	756.5	Intron (NM_001077645, intron 1 of 9)	Prkacb	8.783702048	0.037
chr6	78573556	78573706	+	802.6	Intergenic	Foxa1	8.699413843	0.04
chr15	100333324	100333474	+	710.3	Intron (NM_001107285, intron 2 of 8)	Gpc5	8.593583415	0.046
chr14	95682251	95682401	+	661.9	Intergenic	Cobl	8.488280266	0.051
chr7	42851581	42851731	+	691.9	Intergenic	Kitlg	8.480037172	0.052
chrX	88360079	88,360,229	+	454.3	Intergenic	M6pr	8.457789817	0.053
chr8	42451226	42451376	+	691.9	Intron (NM_198755, intron 6 of 19)	Vwa5a	8.456383844	0.053
chr9	99609362	99609512	+	659.6	Intron (NM_001009825, intron 5 of 9)	Ndufa10	8.42426315	0.055
chr2	64759375	64759525	+	311.4	Intergenic	Cdh6	8.361906849	0.060
chr7	18063362	18063512	+	514.3	Intergenic	Actl9	8.360928233	0.060
chr6	55868585	55868735	+	611.2	Intergenic	Crppa	8.321224094	0.063
chr15	36268860	36269010	+	671.1	Intergenic	Olr1292	8.313645761	0.063
chr7	77691640	77691790	+	627.3	Intron (NM_001011992, intron 1 of 12)	Atp6v1c1	8.275933288	0.067
chr14	47970479	47970629	+	601.9	Intergenic	Wdr82	8.265493331	0.067
chr4	165432453	165432603	+	535.1	Intron (NM_001009486, intron 7 of 9)	Klra1	8.259176271	0.068
chr9	5744478	5744628	+	523.5	Intergenic	Slc5a7	8.251720236	0.068
chr2	172845178	172845328	+	537.4	Intron (NM_001100666, intron 4 of 10)	Schip1	8.247155434	0.069
chr16	66061876	66062026	+	525.8	Intergenic	Unc5d	8.226997253	0.071
chr8	38827590	38827740	+	592.7	Intergenic	LOC688335	8.225052151	0.071
chrX	72411142	72411292	+	472.8	intron (NM_022626, intron 21 of 32)	Hdac8	8.205427729	0.073
chrX	148257277	148257427	+	558.1	Intergenic	Ctag2	8.191302316	0.074
chr1	68760380	68760530	+	581.2	Intergenic	Nlrp4a	8.182958603	0.075
chr9	32913510	32913660	+	599.6	Intergenic	Adgrb3	8.181092592	0.075
chr14	115309174	115309312	+	203	Intergenic	Gpr75	− 5.680445956	0.232
chr2	266405637	266405787	+	493.6	Intron (NM_199408, intron 10 of 12)	Wls	− 4.787204894	0.147
chr13	18217050	18217200	+	66.9	Intergenic	LOC304725	− 2.163526206	0.663
chr6	58515483	58515633	+	267.5	Intron (NM_001163156, intron 6 of 12)	Etv1	− 1.843207673	0.530
chr7	113801451	113801601	+	209.9	Intergenic	Kcnk9	− 1.63483006	0.619
chr5	34879343	34879493	+	154.5	Intergenic	Nkain3	− 1.593760079	0.594
chrX	108951137	108951287	+	292.9	Intron (NM_001166342, intron 3 of 10)	Il1rapl2	− 1.580583359	0.604
chr6	60237696	60237846	+	251.4	Intergenic	Ifrd1	− 1.4524054	0.648
chr15	92593330	92593480	+	106.1	Intergenic	Slitrk1	− 1.395866647	0.744
chr1	74369676	74369826	+	475.1	Intergenic	Vom2r29	− 1.333941331	0.648
chr8	64583723	64583873	+	219.1	Intron (NM_134335, intron 1 of 43)	Myo9a	− 1.308580038	0.699
chrUn	49557	49707	+	290.6	Intergenic	Olr1751	− 1.306983561	0.746
chr8	105859515	105859665	+	267.5	Intron (NM_134377, intron 1 of 17)	Clstn2	− 1.185128373	0.739
chr5	77936229	77936379	+	237.6	Intron (NM_058209, intron 3 of 7)	Zfp37	− 1.184286705	0.723
chr2	123437081	123437231	+	131.5	Intergenic	Trpc3	− 1.16689095	0.752
chr10	32428441	32428591	+	205.3	Intron (NM_001134826, intron 2 of 11)	Sgcd	− 1.153631869	0.723
chr14	113469872	113470022	+	221.4	Intergenic	Pnpt1	− 1.106246452	0.717
chr2	155376169	155376319	+	191.4	Intron (NM_001099506, intron 2 of 4)	Vom2r47	− 1.05925412	0.738
chrX	109671088	109671238	+	110.7	Intron (NM_001166342, intron 7 of 10)	Nrk	− 1.04978499	0.779
chr10	88331010	88331160	+	129.2	Intron (NM_001014120, intron 1 of 9)	Fkbp10	− 1.034412389	0.760
chrX	77496962	77497112	+	62.3	Intron (NM_001191722, intron 23 of 24)	Fndc3c1	− 1.02746036	0.822
chr1	170856786	170856936	+	274.5	Intergenic	Olr214	− 1.010945886	0.748
chr19	42466095	42466245	+	405.9	Intergenic	Pmfbp1	− 1.002170597	0.726
chr6	95271120	95271270	+	203	Intron (NM_001191613, intron 22 of 23)	Pcnx4	− 0.993701348	0.770
chr6	69654982	69655132	+	73.8	Intergenic	Foxg1	− 0.992179761	0.835

Peak calling analysis based on ChIRP probe (uc.104) binding enrichment identified 6282 distinct genomic binding sites of lncRNA uc.104 near protein-coding genes. The distribution of peak scores was visualized using density plots (Fig. 1D), with genes stratified according to their regulation status. Distinct patterns emerged across the categories: upregulated genes displayed a relatively small density peak (slightly > 0.001), with peak scores extending beyond 1000, whereas the non-significant category formed a broader, more pronounced density curve (peaking slightly > 0.005) with peak scores reaching up to 800. No clear enrichment was observed for downregulated genes. Overall, this visualization highlights the separation of regulatory groups, showing that while a small subset of genes exhibited strong upregulation with exceptionally high peak scores, some genes were statistically non-significant and occupied a lower, more diffuse peak score range.

Next, in order to assess reproducibility between ChIRP-seq samples, peak-binding matrices across replicates were compared. Pairwise peak overlap between samples was then quantified using the Jaccard similarity index. CRS rats exhibited moderate (0.36–0.39) overlap of the peak set. This indicates a consistent identification of uc.104 associated binding of genomic loci across samples (Figure S1).

The genomic distribution of these peaks is summarized in Fig. 1E, which shows the proportions mapped to promoter-proximal (± 2 kb from the transcription start site, or TSS), intronic, and distal intergenic regions. The proportion of mapped peaks near intronic and intergenic regions was much higher than in other genomic regions. To determine further whether the genomic distribution of uc. 104 associated peaks reflect specific targeting rather than genomic composition, we normalized the peak distribution to the genomic prevalence of annotation categories. The analysis indicated that in the CRS rat hippocampus, uc.104 associated loci are modestly enriched in intergenic regions and depleted in gene-body regions relative to the genomic background (Figure S2 and Table S2). Next, a Circos plot was created to visualize the genomic distribution of peaks across rat chromosomes (Fig. 1F). The chromosomes were arranged as rainbow-colored ideograms, scaled by their genomic length, with scaffolds smaller than 1 Mb excluded to ensure clarity. This circular layout provided a framework for integrating regulatory information at the genome-wide level. Scatter tracks overlaid on the ideograms highlighted differences in regulation. Upregulated peaks, shown in red, were distributed across multiple chromosomes, with larger points marking regions of particularly high peak scores, indicating chromosomal intervals enriched for strong regulatory activity. In contrast, downregulated peaks, shown in blue, appeared less frequently and were spatially more restricted, pointing to localized sites of repression. Taken together, the visualization revealed an asymmetry in regulatory patterns, with upregulated peaks showing broader genomic coverage and higher intensities, while downregulated peaks were comparatively sparse. The integration of rainbow ideograms with scatter tracks emphasized both chromosomal identity and the relative strength of regulatory changes, providing an intuitive, high-resolution overview of regulatory variation across the rat genome.

Our analysis also indicated that many of the stress-enriched uc.104 binding sites were located at intronic regions harboring promoter elements of 69 protein-coding genes (± 8 kb from TSS). However, based on the literature, we found that 10 (Gabra3, Irs1, Grp37, Htr7, Clu, Hspa1b, Ppp3r2, Nfasc, Pcdhac2, and Cysltr2) of them are implicated in stress neurobiology, synaptic plasticity-related functions, and neuroinflammation. We have presented the data from the ChIRP-seq analysis in Supplemental Table S3.

### Transcriptome profiling of hippocampal gene expression under CRS

**Fig. 2:**
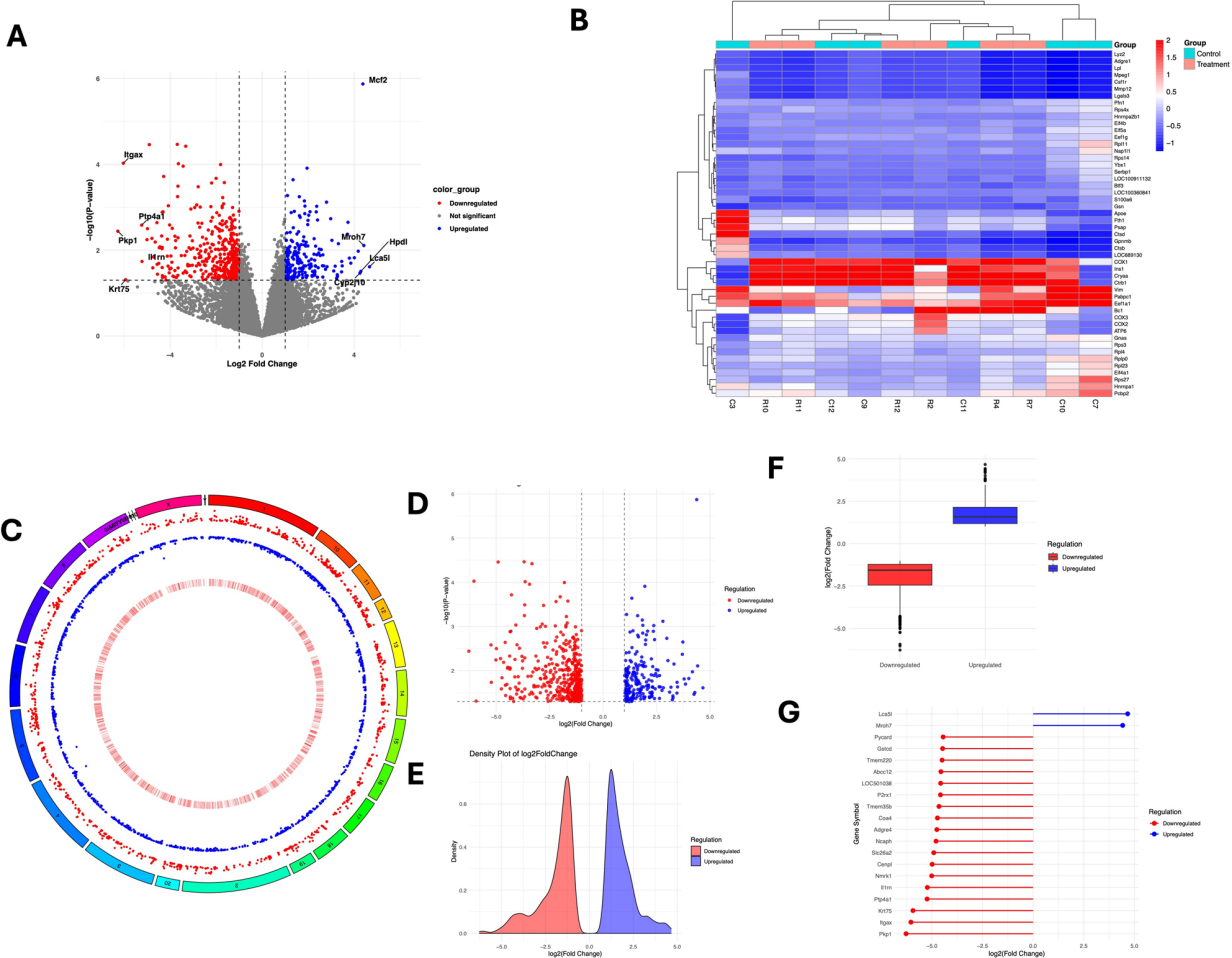
Genome-wide RNA-seq analysis of hippocampal gene expression under chronic restraint stress (CRS). (**A**) Volcano plot showing differentially expressed genes (DEGs) between CRS and control rats. Each dot represents a gene, with significantly upregulated genes in blue, downregulated genes in red, and non-significant genes in grey. (**B**) Heatmap of the top 50 DEGs, with rows representing genes and columns representing samples. High expression is shown in red and low expression in blue. (**C**) Multi-track Circos plot displaying chromosomal distribution of DEGs. The outer ring shows chromosomes in rainbow colors; scatter points indicate upregulated (red) and downregulated (blue) genes positioned by genomic coordinates and fold-change values, while the inner bar track illustrates statistical significance (–log10 p-value) with a yellow-to-red gradient. (**D**) Volcano plot providing a global overview of all DEGs, highlighting their distribution according to log2 fold change (x-axis) and statistical significance (–log10 p-value, y-axis). Upregulated genes are shown in blue and downregulated genes in red. (**E**) Density plot showing the frequency distribution of log2 fold change values. Separate curves are drawn for upregulated genes (blue) and downregulated genes (red), illustrating shifts in expression profiles and overall expression trends under CRS. (**F**) Boxplot comparing log2 fold change values across CRS and control groups, allowing visualization of the overall distribution and magnitude of up- and downregulated genes. (**G**) Lollipop plot of the top 20 DEGs ranked by absolute log2 fold change, with red and blue markers indicating regulation status and stick length corresponding to fold-change magnitude

To investigate genome-wide transcriptional changes in the hippocampus induced by CRS, RNA-seq was performed in hippocampal tissue from control and CRS rats. The sequencing generated high-quality datasets, uniquely aligned to the rat reference genome (Rnor_6.0). In the hippocampus of CRS rats, differential expression analysis identified 14,748 genes after duplicate removal. With a log2FC threshold of ± 1, our analysis identified 266 genes as significantly upregulated and 509 genes as significantly downregulated. The overall distribution of DEGs is illustrated in Fig. 2A as a volcano plot. Each dot represents a single gene, with the x-axis denoting the log2 fold change in expression between CRS and control groups, and the y-axis representing the -log10 of the adjusted p-value. Significantly upregulated genes are highlighted as blue dots, significantly downregulated genes are shown in red, and non-significant genes are displayed in grey.

To further visualize expression patterns and assess clustering between samples, a heatmap was generated by prioritizing genes with high log2FC, assuming that genes with large absolute log2FC are more likely to be biologically relevant. By combining strongly differentially expressed genes and dynamic expression patterns, a set of the top 50 genes was plotted in a heatmap. In the heatmap, each row was scaled with a z-score to achieve row-wise standardization. This heatmap is shown in Fig. 2B, with rows corresponding to individual genes and columns representing biological replicates from control and CRS groups. In the heatmap, genes that are highly expressed are depicted in red, while those with low expression are shown in blue. The top 50 up- and down-regulated genes, ranked by statistical significance and log2FC, are included in Table 2. The table is prepared comprehensively for representing the chromosomal coordinates (including chromosome number, start/end position, and strand specificity) and expression values of all genes.

**Table 2:** Top 50 differentially regulated genes in CRS rat hippocampus

Gene Symbol	Log2 FC	*p*-value	Chr	Start	End	Strand
Lca5l	4.667594517	0.024	11	35461118	35495047	− 1
Mroh7	4.420707058	0.007	5	121463827	121503755	− 1
Mcf2	4.378384684	1.3431E-06	X	138409256	138514446	− 1
Hpdl	4.28792554	0.031	5	130286631	130288233	− 1
Cyp2j10	4.252356892	0.0341	5	110960317	110995115	− 1
Ssmem1	4.177238282	0.010	4	59128730	59145007	1
Pard3b	4.008931913	0.015	9	63038436	64068053	1
Il17rc	3.853529704	0.020	4	146619004	146631442	1
Arhgap28	3.791643991	0.015	9	107833330	107998000	− 1
Kcp	3.73016614	0.002	4	58082857	58109768	− 1
Alox12b	3.705896227	0.004	10	53863060	53874938	1
Zfp605	3.479358203	0.023	12	46545073	46575828	− 1
Grap2	3.459609571	0.018	7	112117142	112190877	1
Crip3	3.452272027	0.032	9	14554450	14557302	− 1
Cyb5d2	3.31419432	0.007	10	57396765	57413715	− 1
Lipt1	3.272433528	0.04	9	40098615	40113565	1
Stac	3.2652233	0.037	8	111530800	111654008	− 1
Sox3	3.237702278	0.045	X	139309329	139310678	− 1
Pkhd1	3.174246084	0.023	9	22549513	23037381	− 1
Gpr137c	3.111238774	0.030	15	18426223	18490807	1
Svep1	3.031617039	0.005	5	72811410	72988525	− 1
Smyd3	2.979197925	0.001	13	90709270	91266253	− 1
Letm2	2.820224304	0.024	16	66469111	66489704	1
Kcna1	2.797759284	0.0007	4	159464188	159472682	− 1
Wdcp	2.753042889	0.027	6	27850698	27865536	1
Pkp1	− 6.275078204	0.003	13	47309614	47357465	− 1
Itgax	− 6.036615273	9.42283E-05	1	182719609	182740698	1
Krt75	− 5.934319705	0.048	7	132688237	132697345	− 1
Ptp4a1	− 5.238887754	0.002	9	33214208	33221964	− 1
Il1rn	− 5.225854273	0.018	3	7111550	7127445	1
Nmrk1	− 5.009309453	0.005	1	216049437	216076791	1
Cenpl	− 4.98828456	0.0031	13	73337257	73352114	1
Slc26a2	− 4.907207529	3.47225E-05	18	54652951	54666626	− 1
Ncaph	− 4.794671357	0.005	3	114371941	114399180	− 1
Adgre4	− 4.754387739	0.025	9	9760355	9900760	1
Coa4	− 4.726100555	0.014	1	154882155	154888174	1
Tmem35b	− 4.649742476	0.006	5	139444399	139449947	1
P2rx1	− 4.57638316	0.002	10	57618586	57633623	1
LOC501038	− 4.566299292	0.021	8	96119602	96128353	1
Abcc12	− 4.553233676	0.036	19	20549405	20620734	1
Tmem220	− 4.496403708	0.020	10	51719777	51728776	1
Gstcd	− 4.472469842	0.008	2	221499083	221591857	− 1
Pycard	− 4.444480676	0.008	1	182601174	182602955	− 1
Hspbap1	− 4.369266824	0.004	11	64940091	64994756	− 1
Slc25a45	− 4.353859353	0.009	1	203166683	203174473	1
Myo18b	− 4.347187861	0.025	12	43747010	43953695	1
Pcgf5	− 4.345671191	0.001	1	234063892	234175315	1
Asf1b	− 4.342424181	0.008	19	24181028	24195549	− 1
Dscc1	− 4.336114785	0.039	7	86482588	86498212	− 1
LOC681367	− 4.333756054	0.043	9	14208417	14208761	− 1

To explore the chromosomal distribution and regulation patterns of DEGs, we generated a multi-track Circos plot from the processed dataset (Fig. 2C). The outermost track displayed chromosomes in a rainbow palette, with lengths inferred from the gene coordinate ranges. Within the plot, two scatter tracks highlighted regulatory patterns: upregulated genes (log2FC ≥ 1) were shown in red and downregulated genes (log2FC ≤ − 1) in blue, each track positioned according to genomic coordinates and fold-change values. A separate bar track illustrated statistical strength using transformed p-values (–log10(P)), with a gradient from light yellow to dark red denoting increasing significance. This integrated visualization provided a genome-wide view of DEG distribution, revealing chromosome-specific clusters and transcriptional hotspots, and enabling simultaneous assessment of both regulation direction and statistical confidence.

To further visualize the expression patterns of up- and downregulated genes, we generated several complementary plots (Fig. 2D-G). A volcano plot provided a global overview of gene distribution by effect size (log2FC) and statistical significance (–log10 p-value), with upregulated genes highlighted in blue and downregulated genes in red (Fig. 2D). This representation enabled the simultaneous identification of significantly altered genes and their regulation direction. To further assess distribution patterns, a boxplot compared log2FC values across the two groups, while a density plot illustrated the overall distribution profiles, revealing shifts in expression trends between up- and downregulated sets (Fig. 2E & F). Finally, a lollipop plot focused on the top 20 genes ranked by absolute log2FC shows their regulation status and the magnitude of differential expression (Fig. 2G). Collectively, these plots provided complementary perspectives that highlighted both global and gene-specific transcriptional changes. The data generated from the differential gene expression analysis have been provided in Supplemental Table S4.

### Identification of lncRNA-regulated gene networks using integrated ChIRP-seq and RNA-seq data

**Fig. 3:**
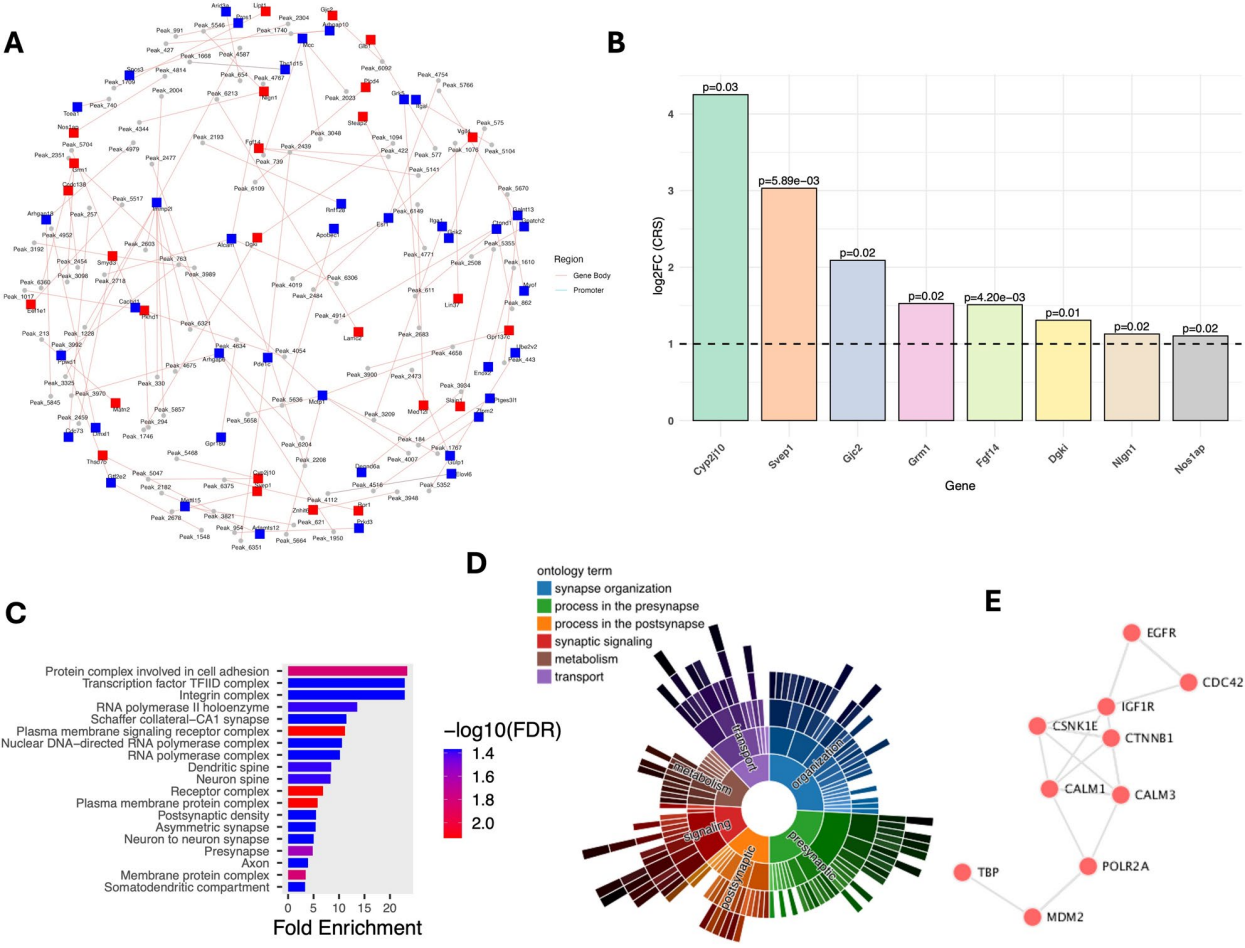
Integration of uc.104 ChIRP-seq and RNA-seq data with functional annotation. (**A**) Cytoscape network showing the overlap between uc.104 binding peaks and differentially expressed genes (DEGs) in the hippocampus of CRS rats. Nodes represent genes and edges indicate uc.104–gene associations. (**B**) Differential expression profile of select genes between CRS and control rats are shown with a bar diagram. The diagram illustrates the normalized log₂FC (fold change) in expression levels for selected genes in the CRS group. Each bar is represented with a single gene, with unique color-coding for visual clarity. A dashed horizontal line at log₂FC = 1 marks the control reference level. The log₂FC values are based on the mean of 6 rats from each group. The values above bars denote statistical significance based on p-values (< 0.05) obtained from differential expression analysis. All genes showing *p* < 0.05 are considered significantly differentially expressed. (**C**) Bar plot of the top 20 enriched Gene Ontology (GO) biological processes for uc.104-bound DEGs. The x-axis shows –log10 adjusted p-values, and bar colors reflect the gene ratio within each term. (**D**) Sunburst plot from SynGO illustrating synapse-related processes associated with uc.104 target genes, including pre- and postsynaptic organization, signaling, and metabolism. (**E**) Protein–protein interaction (PPI) network generated using STRING, highlighting 10 hub genes (EGFR, CDC42, IGF1R, CTNNB1, CALM1, CALM3, POLR2A, MDM2, TBP, and CSNK1E). Node size corresponds to network centrality, and connections denote high-confidence interactions

To investigate the potential regulatory roles of uc.104 in modulating hippocampal gene expression under CRS, we integrated genome-wide lncRNA binding profiles obtained from ChIRP-seq with DEGs identified from RNA-seq analysis. This integration was performed by intersecting the genomic locations of lncRNA binding peaks with the annotated promoters (± 2 kb from TSS) and gene bodies of DEGs. The results of this intersection are illustrated in Fig. 3A, which presents a lncRNA–gene interaction network constructed and visualized using Cytoscape. The network, depicting the overlap between enriched genes associated with uc.104 ChIRP-seq peaks and the set of DEGs identified in the CRS hippocampus. A total of 1839 DEGs were associated with at least one lncRNA binding site, suggesting that these genes are likely direct targets of uc.104 (Table S5). After applying statistical significance (*p* ≤ 0.05), 106 overlapping genes were determined in close proximity to the uc.104 binding sites (Table S6).

### Functional annotation of lncRNA-bound DEGs

To further explore the potential targets of the upregulated long non-coding RNA uc.104, an analysis was performed to identify peaks located near protein-coding genes. From 6,664 peaks in the dataset, 621 peaks were classified as upregulated (log2FC > 1) and found within ± 25 kb of protein-coding regions (Table S7). This subset represents the candidate genomic loci that could be influenced by uc.104-mediated regulation. The strongest associations were observed near several genes with high fold-change values. The top 10 genes ranked by log2FC included Foxa1 (8.699), Vwa5a (8.456), Crppa (8.321), Olr1292 (8.314), Atp6v1c1 (8.276), Olr404 (8.076), Agpat3 (8.052), Rpl13 (8.041), Hyal4 (8.041), and Myo16 (8.010). These genes represent the most prominent protein-coding candidates that are targeted by upregulated peaks associated with uc.104 activity. Additionally, in Fig. 3B, we have presented the expression profiles of 8 prominent genes (Dgki, Svep1, Nlgn1, Nos1ap, Cyp2j10, Fgf14, Gjc2, and Grm1) for their potential role in stress. The eight genes have shown significant upregulation in CRS rats and are mapped to genomic coordinates intersected by uc. 104 binding.

To gain insight into the biological roles of DEGs overlapping with uc.104 ChIRP-seq peaks, a gene ontology (GO) enrichment analysis was performed. The results are summarized in Fig. 3C, which illustrates the top 20 significantly enriched GO biological processes (BP) for each gene set. In Fig. 3C, the bar plot displays the enriched GO terms for genes upregulated in CRS animals. The x-axis represents the -log10 of the adjusted p-value, indicating the statistical significance of each term, while the y-axis lists the biological processes. The bars are colored according to gene ratio, defined as the proportion of DEGs associated with a given GO term relative to the total number of genes in that term. The analysis revealed strong enrichment of upregulated genes in processes related to dendritic spine, neuronal spine formation, presynapse, neuron-to-neuron synapse formation, and axonal changes. Additional enrichment was observed in processes associated with membrane protein complex formation and somatodendritic compartment formation (Table S8).

Using the SynGO (Synaptic Gene Ontology) database, further mapping of the promoter proximal genes potentially operated by uc.104 binding revealed ontological terms highly enriched for synapse organization, affecting processes in pre- and postsynaptic functions, and synaptic signaling, with additional enrichment to synaptic metabolism and transport. This result is illustrated in Fig. 3D as a sunburst plot. These findings indicate the role of uc.104 as a facilitator of stress-induced gene expression changes affecting synapse functionality in the hippocampus. Based on their previously reported roles in the neurobiology of stress, we identified three distinct sets of genes associated with stress/HPA (hypothalamus-pituitary-adrenal axis), neuroinflammation, and synaptic plasticity. A separate table is provided for this (Table S).

Finally, the significantly affected overlapping genes regulated by uc.104 were analyzed using the STRING database to construct a core protein–protein interaction (PPI) network (Fig. 3E). This analysis identified 10 hub genes — EGFR, CDC42, IGF1R, CTNNB1, CALM1, CALM3, POLR2A, MDM2, TBP, and CSNK1E. Notably, several of these are strongly linked to stress responses and neurobiology: EGFR and IGF1R are central mediators of stress-responsive signaling cascades; CDC42 and CTNNB1 play key roles in cytoskeletal reorganization and neuronal plasticity under stress conditions; while CALM1 and CALM3 highlight calcium-dependent pathways critical for neurobiological stress responses. These findings suggest that uc.104 may influence neuronal stress-responsive mechanisms by targeting hub genes at the intersection of stress and other neurobiological pathways.

## Discussion

The present study examined the genome-wide chromatin occupancy of the stress-responsive lncRNA uc.104 in the hippocampus of CRS rats. ChIRP-seq experiment using uc.104-specific probes produced high-quality datasets with high confidence mapping to the rat genome. This demonstrates the specificity and robustness of the method for detecting lncRNA–chromatin interactions. Stress induces widespread transcriptional and epigenetic alterations in the hippocampus, and lncRNAs have emerged as important regulators in this process. Previous reports have shown that several brain-expressed lncRNAs, such as Bdnf-AS and Gomafu, regulate synaptic genes and stress reactivity [34, 35]. Our findings in the CRS rats add uc.104 to this group by showing that its chromatin occupancy changes markedly under chronic restraint stress.

It is pertinent to mention that this study was designed as an exploratory analysis aimed at identifying candidate genomic loci associated with uc.104 in the hippocampus of the CRS rat brain. We also acknowledge that the sample size was small, a common practical limitation in the ChIRP pulldown experiment due to the assay’s material requirements. Thus, the differential binding results were interpreted conservatively and integrated with independent datasets (intersected with RNA-seq results and functional enrichment analyses) to prioritize biologically relevant gene promoters and regulatory elements in the genome. We found that uc.104 is a significantly and consistently elevated lncRNA in the hippocampus of CRS rats, supporting its role in stress responsiveness. Moreover, this uniformity across different stress models is further validated by our previous results in the learned helplessness rat model, where we also observed significant upregulation of this lncRNA in LH rat hippocampus [14]. Our ChIRP-seq results following uc.104-chromatin touch point analysis suggested more than 6,000 stress-enriched uc.104 peaks, with the strongest signals near genes such as Foxa1, Prkacb, and Mff. Each of these genes has previously been associated with transcriptional regulation, kinase signaling, or mitochondrial function in the hippocampus, processes that are consistently affected by chronic stress [36–40]. Thus, uc.104 appears to bind selectively at stress-sensitive loci. It has been noted that the majority of uc.104 binding sites were located in intronic or intergenic regions rather than promoter-proximal sequences. This is consistent with earlier observations showing that lncRNAs often associate with distal regulatory elements and enhancer-like regions [41]. The asymmetric distribution across the genome, with more enriched than reduced peaks in our peak analysis, also mirrors observations that stress exposure tends to enhance the activity of inducible regulatory complexes in the hippocampus [7]. As acknowledged earlier, our ChIRP-seq dataset is an exploratory analysis; however, interestingly, replicate concordance analysis based on peak overlap indicated consistent identification of uc. 104 binding across genomic loci and across samples. These results support the robustness of the detected peak binding signals and emphasize the need for future studies with an expanded replication cohort. Our analysis identified stress-enriched uc.104 binding near 10 protein-coding genes with established roles in stress neurobiology and synaptic plasticity. Gabra3 and Htr7 encode neurotransmitter receptors critical for inhibitory and serotonergic signaling, both of which are targets of stress-related plasticity in hippocampal circuits [42]. Irs1 integrates insulin/IGF signaling with neuronal stress responses, while Gpr37 has been implicated in dopaminergic neuroprotection [43]. Heat shock proteins (Hspa1b, Clu) represent classical stress-induced chaperones, and Ppp3r2, Nfasc, and Pcdhac2 are required for synaptic organization and activity-dependent remodeling [44–46]. Cysltr2 links uc.104 binding to inflammatory pathways, which are increasingly recognized as modulators of stress pathology [47–51]. These findings suggest that uc.104 may contribute to hippocampal stress adaptation by interacting with chromatin near genes that coordinate neurotransmission, synaptic remodeling, and inflammatory signaling [18, 52–55].

Previous reports suggested that chronic stress can induce widespread transcriptional remodeling in the hippocampus [56], with large sets of differentially expressed genes involved in synaptic signaling, neuroplasticity, and stress hormone pathways [57, 58]. Many of these changes are associated with underlying structural reorganization, a hallmark of mood and cognitive disturbances [59–61]. In agreement with this literature, our RNA-seq analysis revealed 775 significantly altered transcripts in the hippocampus of CRS rats, with a notable proportion of genes upregulated. The predominance of upregulated genes is consistent with evidence that chronic stress induces maladaptive changes in gene regulation, disturbs hippocampal activity, and alters neuroplasticity-related transcription.

It is worth noting that our data visualization using heatmaps confirmed clustering of stress-responsive genes, with substantial fold changes observed in subsets of both upregulated and downregulated transcripts. It is also interesting to note that the genes showing the most pronounced regulation are linked to neurotransmitter systems, intracellular signaling, and metabolic function [62]. This pattern aligns with previous reports indicating that stress alters glutamatergic and GABAergic signaling, intracellular kinase cascades, and mitochondrial pathways in the hippocampus [63–65]. Circos visualization further demonstrated that the differentially expressed genes were not evenly distributed but formed clusters on specific chromosomes. Chromosomal hotspots of transcriptional change have been observed in other RNA-seq studies of stress, and reflect coordinated regulation of gene families or local chromatin domains responsive to glucocorticoids and other stress mediators [66]. The integrated scatter and bar tracks provided a genome-wide view of both regulatory direction and statistical significance, enabling the identification of regions with concentrated transcriptional remodeling. Complementary plots, including density and box plots, showed global shifts in expression patterns, while the lollipop plot highlighted the top 20 most strongly regulated genes. Many of these genes overlapped with molecular pathways previously implicated in synaptic plasticity, neuronal excitability, and neuroimmune interactions, supporting the view that CRS alters hippocampal networks through multiple convergent mechanisms [67]. Taken together, our results provide a transcriptomic framework that complements our chromatin occupancy findings for uc.104, and suggest that lncRNA-mediated regulation may contribute to the transcriptional reprogramming observed under chronic stress [68].

As we sought to integrate ChIRP-seq and RNA-seq data, this integration provided meaningful insights into the regulatory functions of uc.104 in the hippocampus under CRS. By intersecting uc.104 chromatin occupancy with differentially expressed genes, we identified 1,839 genes with at least one binding site, suggesting widespread potential for transcriptional regulation. After applying a stringent significance threshold, 106 genes remained as high-confidence overlaps, strongly supporting direct regulatory interactions between uc.104 and stress-responsive genes. The convergence of lncRNA binding and transcriptional change is consistent with the proposed role of lncRNAs as modulators of gene expression through chromatin-associated mechanisms [69–71]. Prior studies have shown that lncRNAs can recruit chromatin modifiers, interact with transcription factors, and influence RNA polymerase activity at specific loci [70, 72, 73]. In this context, the overlap between uc.104 binding and differentially expressed genes indicates that this lncRNA may exert direct effects on transcriptional programs that are remodeled under chronic stress [12].

The magnitude of overlap also underscores the functional importance of uc.104 in hippocampal stress biology [71]. Previous RNA-seq studies have reported widespread suppression of plasticity-related transcripts and induction of stress-response genes in chronic stress models [74, 75]. Our integrative approach suggests that uc.104 may be one of the molecular regulators orchestrating these changes by directly binding to promoter regions and gene bodies of stress-responsive loci [76]. This combined analysis, thus, places uc.104 at the intersection of chromatin regulation and transcriptional control in the stressed hippocampus. The set of 106 high-confidence overlapping genes provides a focused group of candidates for further functional studies aimed at clarifying how uc.104 contributes to neuronal adaptation and vulnerability under chronic stress conditions [77].

Identification of uc.104 binding peaks near protein-coding genes provides further insight into its potential role in regulating transcriptional programs under chronic stress. Out of 6,664 peaks detected, 621 were classified as upregulated within ± 25 kb of protein-coding loci, highlighting genomic regions most likely influenced by uc.104 activity. The strongest associations were observed near genes such as Foxa1, Vwa5a, Crppa, Olr1292, Atp6v1c1, and Myo16, which represent potential direct targets of stress-induced uc.104 binding [8, 78]. While several of these genes are not yet well characterized in stress contexts, strong fold changes underscore their potential as candidate effectors of lncRNA-mediated regulation. GO enrichment analysis of differentially expressed genes overlapping uc.104 binding peaks revealed significant enrichment for processes associated with synaptic organization and plasticity [79]. Terms included dendritic spine and presynapse formation, neuron-to-neuron synapse development, axonal remodeling, and membrane protein complex assembly [80]. These findings are consistent with prior evidence that chronic stress alters dendritic morphology and synaptic connectivity in the hippocampus. Analysis using the SynGO database further supported this interpretation, demonstrating enrichment of uc.104-associated genes in both pre- and postsynaptic compartments, synaptic signaling, and synaptic metabolism [81]. These results suggest that uc.104 may contribute to stress-induced reorganization of hippocampal circuits through regulation of synaptic genes [82]. Furthermore, independent classification of the overlapping genes according to previous literature enabled the identification of distinct subsets related to HPA axis regulation, neuroinflammation, and synaptic plasticity [83]. This functional grouping reinforces the notion that uc.104 operates at the intersection of stress signaling, immune responses, and synaptic remodeling — processes that are central to hippocampal adaptation and vulnerability to stress-related disorders [62].

Protein–protein interaction analysis additionally highlighted hub genes that may serve as key regulatory nodes influenced by uc.104. Among these, EGFR and IGF1R are well established as upstream regulators of stress-related signaling pathways [84–86]. Genes such as CDC42 and CTNNB1 contribute to cytoskeletal dynamics and synaptic structure, while CALM1 and CALM3 represent calcium-binding proteins that are crucial for synaptic signaling and neuronal stress adaptation [87]. MDM2 is a central regulator of p53-mediated stress responses, and POLR2A and TBP point to transcriptional machinery as potential downstream effectors. These hub genes place uc.104 at the convergence of pathways governing neuronal stress responses, structural plasticity, and transcriptional control [7, 88, 89].

Although our study is one of the first to report lncRNA-associated gene regulatory changes potentially mediated by chromatin modification, it has certain limitations [88]. For example, our ChIRP-seq findings identified genomic loci associated with uc.104 binding. While this pattern suggests a role for uc.104 in chromatin-associated transcriptional changes, further studies are needed to determine whether uc.104 influences gene transcription at these sites. More specifically, it requires future functional work to test whether uc.104 directly regulates transcription at these sites and whether its modulation alters behavioral outcomes of chronic stress. To be more precise, understanding of any alteration in the chromatin induced by the binding of uc.104 at the level of histone acetylation or methylation will be the key. Nevertheless, our results indicate that uc.104 is positioned to influence hippocampal stress adaptation by binding to chromatin near synaptic and stress-responsive genes, and by targeting central nodes within protein–protein interaction networks. This integrative regulatory role suggests that uc.104 may act as a mediator linking chronic stress exposure to transcriptional reorganization in the hippocampus.

## Supplementary Information

Below is the link to the electronic supplementary material.

Supplementary Material 1: Figure S1: Replicate concordance analysis of ChIRP-seq samples. Pairwise peak overlap between samples was quantified using the Jaccard similarity index and visualized as a clustered heatmap.

Supplementary Material 2: Figure S2: Normalized enrichment of uc.104 associated ChIRP-seq peaks across genomic annotations.

Supplementary Material 3

Supplementary Material 4

Supplementary Material 5

Supplementary Material 6

Supplementary Material 7

Supplementary Material 8

Supplementary Material 9

Supplementary Material 10

Supplementary Material 11

## Data Availability

All data generated or analyzed during this study are included in this published article [and its supplementary information files].
