# Supplementary figures and images for: Genome-wide mapping of stress-responsive lncRNA, uc.104, reveals the chromatin-mediated regulation of stress and plasticity-related genes in the hippocampus of chronic restraint rats
Source: Mol Brain. 2026 Apr 19;19:43. doi: 10.1186/s13041-026-01304-3 (PMC13220396; doi:10.1186/s13041-026-01304-3)

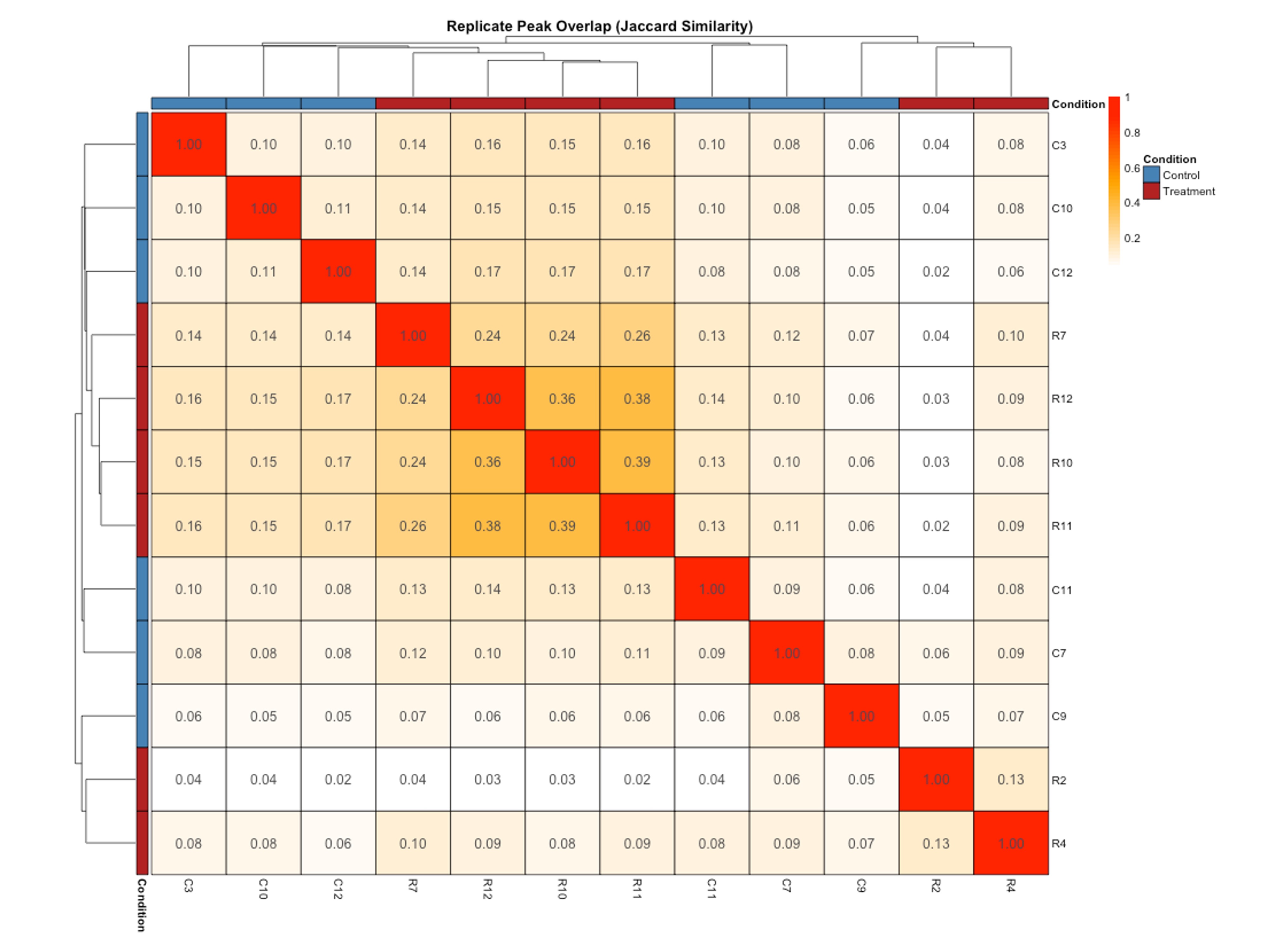

Supplement: Supplementary file 1 — Supplementary Material 1: Figure S1: Replicate concordance analysis of ChIRP-seq samples. Pairwise peak overlap between samples was quantified using the Jaccard similarity index and visualized as a clustered heatmap. [file 13041_2026_1304_MOESM1_ESM.jpeg]

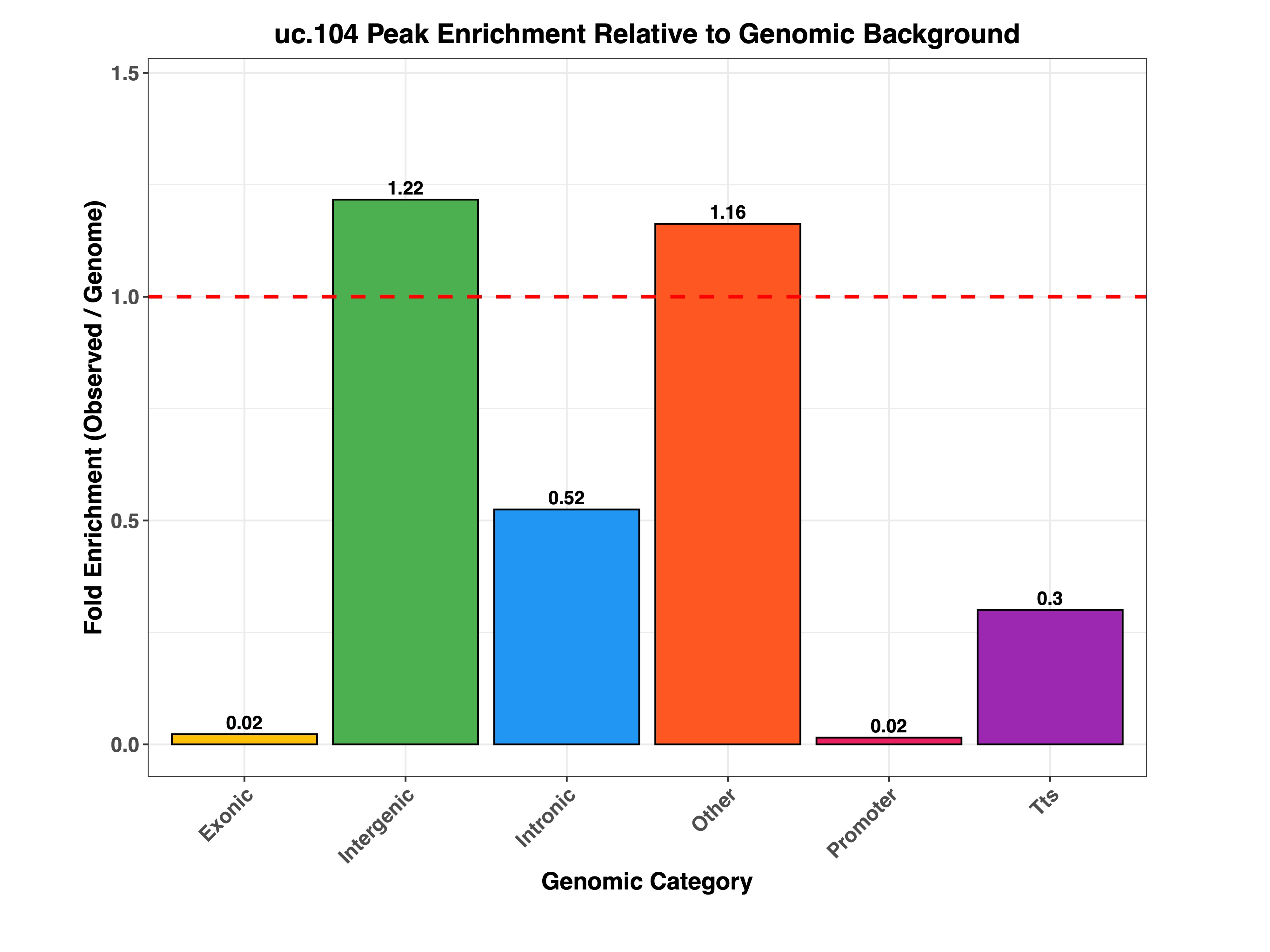

Supplement: Supplementary file 2 — Supplementary Material 2: Figure S2: Normalized enrichment of uc.104 associated ChIRP-seq peaks across genomic annotations. [file 13041_2026_1304_MOESM2_ESM.jpeg]
